# Development and Validation of Multivariable Prediction Models for In-Hospital Death, 30-Day Death, and Change in Residence After Hip Fracture Surgery and the “Stratify-Hip” Algorithm

**DOI:** 10.1093/gerona/glad053

**Published:** 2023-02-09

**Authors:** Aicha Goubar, Finbarr C Martin, Catherine Sackley, Nadine E Foster, Salma Ayis, Celia L Gregson, Ian D Cameron, Nicola E Walsh, Katie J Sheehan

**Affiliations:** School of Life Course and Population Sciences, Faculty of Life Science and Medicine, King’s College London, London, UK; School of Life Course and Population Sciences, Faculty of Life Science and Medicine, King’s College London, London, UK; School of Life Course and Population Sciences, Faculty of Life Science and Medicine, King’s College London, London, UK; Surgical Treatment and Rehabilitation Service (STARS) Education and Research Alliance, The University of Queensland and Metro North Health, Brisbane, Queensland, Australia; Primary Care Centre Versus Arthritis, School of Medicine, Keele University, Keele, UK; School of Life Course and Population Sciences, Faculty of Life Science and Medicine, King’s College London, London, UK; Musculoskeletal Research Unit, Translation Health Sciences, Bristol Medical School, University of Bristol, Bristol, UK; John Walsh Centre for Rehabilitation Research, Northern Sydney Local Health District and University of Sydney, Ryde, New South Wales, Australia; Centre for Health and Clinical Research, University of the West of England Bristol, Bristol, UK; School of Life Course and Population Sciences, Faculty of Life Science and Medicine, King’s College London, London, UK

**Keywords:** Classification, Fracture neck of femur, Fragility fracture, Recovery, Stratification

## Abstract

**Background:**

To develop and validate the stratify-hip algorithm (multivariable prediction models to predict those at low, medium, and high risk across in-hospital death, 30-day death, and residence change after hip fracture).

**Methods:**

Multivariable Fine-Gray and logistic regression of audit data linked to hospital records for older adults surgically treated for hip fracture in England/Wales 2011–14 (development *n* = 170 411) and 2015–16 (external validation, *n* = 90 102). Outcomes included time to in-hospital death, death at 30 days, and time to residence change. Predictors included age, sex, pre-fracture mobility, dementia, and pre-fracture residence (not for residence change). Model assumptions, performance, and sensitivity to missingness were assessed. Models were incorporated into the stratify-hip algorithm assigning patients to overall low (low risk across outcomes), medium (low death risk, medium/high risk of residence change), or high (high risk of in-hospital death, high/medium risk of 30-day death) risk.

**Results:**

For complete-case analysis, 6 780 of 141 158 patients (4.8%) died in-hospital, 8 693 of 149 258 patients (5.8%) died by 30 days, and 4 461 of 119 420 patients (3.7%) had residence change. Models demonstrated acceptable calibration (observed:expected ratio 0.90, 0.99, and 0.94), and discrimination (area under curve 73.1, 71.1, and 71.5; Brier score 5.7, 5.3, and 5.6) for in-hospital death, 30-day death, and residence change, respectively. Overall, 31%, 28%, and 41% of patients were assigned to overall low, medium, and high risk. External validation and missing data analyses elicited similar findings. The algorithm is available at https://stratifyhip.co.uk.

**Conclusions:**

The current study developed and validated the stratify-hip algorithm as a new tool to risk stratify patients after hip fracture.

The age-standardized rate of hip fracture ranges from lows of 2/100 000 in Nigeria (women) and 35/100 000 in Ecuador (men), to highs of 574/100 000 in Denmark (women) and 290/100 000 in Denmark (men) (1). Even with surgery, up to 10% of patients die in hospital, and 22% transition from living at home to care homes (2). Multidisciplinary and orthogeriatric-led management is the optimal approach for acute hospital care after hip fracture, resulting in fewer deaths and transitions to care homes (risk ratio 0.88, 95% confidence interval [CI] 0.80, 0.98) (3). Early and frequent therapy input is also associated with an additional 2% of patients returning home and 4% of patients surviving to 30 days (4). Yet, a demand–capacity mismatch often limits the delivery of consistent orthogeriatric care (5) and therapy services in hospital after hip fracture (6). This mismatch requires clinicians to prioritize their caseload based on perceived need. However, variation in national audit data (earlier what may be explained by differences in case mix) may suggest a lack of consistency in this prioritization (5,6).

A stratified approach to multidisciplinary care delivery may improve efficiency and reduce inconsistencies in prioritization by identifying groups of patients at risk of poor outcomes to be matched to different treatments, acknowledging different needs and potential benefits from healthcare professional input. To achieve this, prediction models are necessary. Most previously published models have limitations in performance and/or implementation (7). In contrast, the Nottingham Hip Fracture Score has modest discrimination and adequate calibration for death and includes predictors which clinicians can collect prior to surgery (8). The Nottingham Hip Fracture Score was developed to inform consenting procedures, timing of surgery, access to pre/postoperative higher level care, and audit (8). However, it was not designed to enable the stratification of patients into different risk groups to be matched to different treatments.

This study aimed to develop and validate the stratify-hip algorithm (comprised of 3 multivariable prediction models) using routinely collected data available on admission as a new tool to risk stratify patients after hip fracture. The algorithm sought to predict those at low, medium, and high risk across time to in-hospital death, death by 30 days, and time to change in residence, and to be able to discriminate between the groups. The stratify-hip algorithm and link to a freely available web-based app to facilitate risk prediction are provided.

## Method

This study is reported according to the transparent reporting of a multivariable prediction model for individual prognosis or diagnosis (TRIPOD) statement (9). The study did not require ethical approval as it involved secondary analysis of pseudonymized data.

### Source of Data

The UK National Hip Fracture Database (NHFD) collates data on the characteristics of 95% of patients aged 60 years and older with hip fracture and the care they received during their acute hospital stay in England and Wales (5). Individual patient NHFD data were linked to electronic hospital records and the Office of National Statistics records for data on dementia diagnosis and death, respectively. Further details on data cleaning and linkage across databases are described elsewhere (10). Data submitted to the NHFD for 170 411 patients surgically treated for a nonpathological first hip fracture between January 1, 2011, and December 31, 2014, were selected for development and internal validation (follow-up to 30-day post-admission or to December 31, 2014). Data submitted for 90 102 patients treated between January 1, 2015, and December 31, 2016, were selected for external (temporal) validation (follow-up to 30-day post-admission or to December 31, 2016). Differences between patients with and without complete predictor data are presented in Supplementary Tables 1 and 2. Those with missing predictor data were more dependent (greater proportion with dementia [all outcomes] and admitted from nursing/residential care [in-hospital death and 30-day death]) than those without missing data.

### Outcomes

Outcomes included (a) time to in-hospital death, (b) death status at 30 days post-admission, and (c) time to change in residence (among those admitted from home) as key performance indicators of safe and effective care (5), and which reflect the patient priority of returning home (11). Time to in-hospital death was calculated as the number of days from admission to a coded discharge destination of death, treating discharge to another unit (loss to follow-up), or to Day 30 (end of follow-up) as a censoring event, and discharge home or to nursing/residential care as a competing event. Thirty-day death was identified by a binary indicator (alive, dead) 30 days post-admission. Time to change in residence (pre-fracture residence of home and discharge destination of nursing/residential care, Supplementary Table 3) was calculated as the number of days from admission to a residence change, treating discharge to another unit (loss to follow-up), or to Day 30 (end of follow-up) as a censoring event, and return to pre-fracture residence or in-hospital death as competing events.

### Predictors

Five predictors were included: age (5-year age groups from age 60 years at admission), sex (male, female), pre-fracture mobility (no functional mobility, independent indoor mobility with/without aid, independent outdoor mobility with/without aid), dementia diagnosis (International Classification of Diseases 10th Edition code F00, F01, F02, F03, or G30 during the hip fracture admission or any admission in the year prior), and pre-fracture residence (own home/sheltered housing, nursing/residential care [not for time to change in residence outcome]). Predictors were defined a priori following evidence review (2,12), interviews with patients (11) and healthcare professionals (13,14), a public and patient involvement focus group, and which were available in the data set. To optimize the simplicity of future implementation of risk stratification, the number of predictors was kept to a minimum and to those which could be feasibly collected preoperatively by any healthcare professional either directly from the patient or an informal/formal carer.

### Sample Size

The similarity between the external and development data sets was anticipated in terms of the distribution of patients by each predictor. The extent to which the external validation data set sample size could be justifiable to perform a validation of our models was assessed. For the observed proportion of 30-day deaths (5.8%), an estimated minimum of 13 751 patients (with 798 deaths at 30 days) was required for (a) a target standard error (*SE*) of the logarithm (observed/expected [O/E] ratio) of 0.245 with a target O/E ratio of 1; (b) a target area under the receiver-operating characteristic curve (AUC) of 0.70 with *SE*(AUC) of 0.0225; and (c) and a target calibration slope of 1 with an *SE* of 0.051 (15).

### Statistical Analysis Methods

Predictors were described by counts and proportions.

#### Time-to-event outcomes

Fine-Gray (16) regression was used to build a prediction model estimating the direction of the association between predictors and the cumulative incidence (risk) of in-hospital death and of residence change as functions of postoperative day, accounting for competing events (17). Discharges to another care setting and hospital stays exceeding 30 postoperative days were treated as right-censored observations. The model was fitted with R (18) software using *RiskRegression* packages (19) and *cmprsk* (20). The proportional hazards assumption was assessed by plotting Schoenfeld residuals against failure time with a scatterplot smoother for each covariate in the models (21). Model calibration was assessed by estimating the O/E ratio and plotting the mean predicted risk against observed risk for predicted risk deciles at 30 days from admission (22). Model discrimination was assessed by C-index statistics at 30 days (or AUC) (23). The Brier score was also calculated as the expected squared distance between predicted and observed risk at 30 days from admission (24). This score accounts for both calibration and discrimination with a lower score (scale 0%–100%) indicating a higher performing model (25).

#### Binary outcome

A 5-predictor logistic regression model was used to predict the risk of death at 30 days. The presence of influential observations was examined by visualizing the Cook’s distance values (26). Multicollinearity among predictors was investigated using “vif” in the “rms” R package, which computes the variance inflation factors (VIF) (27,28). Model calibration was assessed by quantifying the calibration slope and the O/E ratio (29,30). The mean predicted risk against observed risk for predicted risk deciles (22) was also plotted and a generalized additive model with integrated smoothness was presented (31). Discrimination was assessed by the C statistic (29) and both calibration and discrimination by the Brier score (29). The analyses were conducted with R (18) using *rms* packages (32).

#### Internal validation

For internal validation, 100 bootstrap samples were generated with replacement from the development data set (33). Each sample included the same number of patients as the development data set. Performance was assessed from each bootstrap model in each bootstrap sample (apparent performance) and the performance of each bootstrap model in the development dataset (test performance) (34). Optimism (overestimation bias often due to overfitting) was calculated as the average difference between apparent and test performance across bootstrap samples (34). Optimism-adjusted measures of performance (AUC and Brier scores) were estimated by subtracting the estimate of optimism from the development model performance estimates (34).

#### Risk groups

**Figure 1.**
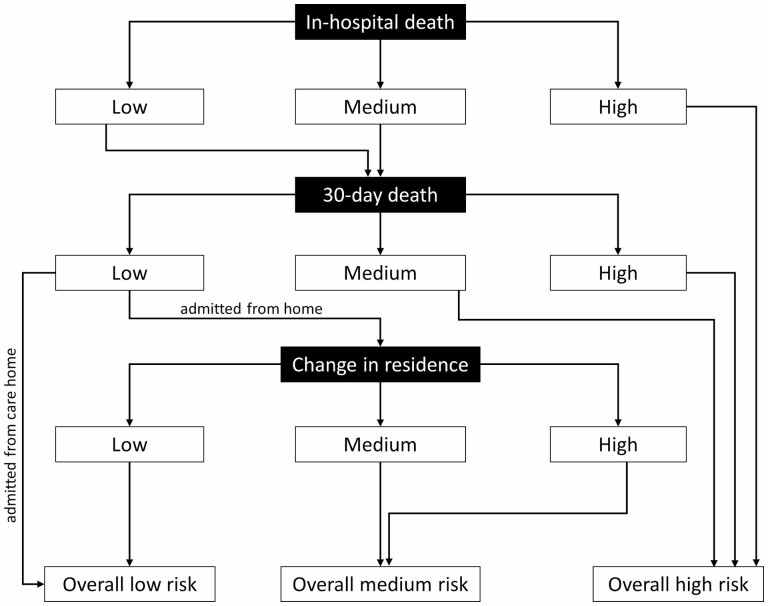
Stratify-hip algorithm to enable patient assignment to 3 overall risk groups based on predicted risk of in-hospital death, 30-day death, and change in residence.

Patients were clustered into 3 risk groups (low, medium, and high) for each outcome by applying *K*-means clustering algorithm (an algorithm which partitions n observations into *k* clusters in which each observation belongs to the cluster with the nearest mean) (35). The number of groups was defined a priori to balance risk assignment with the feasibility of designing future matched treatments for each risk group. Following clustering for each outcome, patients were assigned to 1 of 3 groups: overall low (low risk across outcomes), overall medium (low risk of death but medium or high risk of change in residence), and overall high (high risk of in-hospital death, high or medium risk of 30-day death) risk across outcomes (Figure 1).

#### Sensitivity analyses

Multiple chained equations (MICE) were used to determine the sensitivity of findings to data missingness (36). Twenty distinct data sets were generated for efficient and stable estimates (36). Missing predictor values were replaced iteratively with values from multiple regression models within the MICE in addition to auxiliary variables Charlson comorbidity index, American Society of Anaesthesiologists Classification, deprivation (Index of Multiple Deprivation decile groups), and type of surgery (arthroplasty, hemiarthroplasty, internal fixation) to minimize bias and optimizes the power of the imputations (37). As in the main analysis, either Fine-Gray or logistic regression models were used, as appropriate, to predict the risk of each outcome. The optimism corrected values of AUC and Brier score for each model within the 20 imputed data sets were estimated prior to the generation of pooled values and their CIs across imputed data sets (38).

The influence of age groups on model performance was assessed by treating age as a continuous variable in sensitivity analyses of the development data set.

#### External validation

The 3 models generated in the development data set were applied to the external validation data set to estimate the predicted risk. Performance was estimated through the AUC and Brier scores. Risk groups were subsequently generated as described earlier.

#### Model access

The final model is accessible via a freely available web-based app. Nomograms were also generated for each outcome to be used alongside Figure 1 for settings where internet access is not available (39). Nomograms were generated with R (18) packages *rms* (32) and *cmprsk* (20).

## Results

### Participants

Among 170 411 patients, 141 158 (83%), 119 420 (70%), and 149 258 (88%) had complete data for predictors and in-hospital death, 30-day death, and change in residence, respectively. The majority were women, admitted from home, and were able to ambulate outdoor pre-fracture (Table 1). More than half were over 80 years of age, and one-quarter had a diagnosis of dementia.

**Table 1.** Characteristics of Patients Surgically Treated for Nonpathological First Hip Fracture for Development and Internal Validation, and External Validation Data Sets

		In-Hospital Death		30-Day Death		Change in Residence^d^	
		Development and Internal Validation (2011–14)	External Validation 2015-16)	Development and Internal Validation (2011–14)	External Validation (2015–16)	Development and Internal Validation (2011–14)	External Validation (2015–16)
		*n* =141 158	*n* = 84 096	*n* = 149 258	*n* = 87 414	*n* = 119 420	*n* = 70 319
Age at admission (y)	60–64	4 618 (3.3)	2 641 (3.1)	4 910 (3.3)	2 772 (3.2)	4 616 (3.9)	2 586 (3.7)
	65–69	7 867 (5.6)	5 108 (6.1)	8 348 (5.6)	5 346 (6.1)	7 711 (6.5)	4 910 (7.0)
	70–74	11 453 (8.1)	7 239 (8.6)	12 117 (8.1)	7 560 (8.6)	10 965 (9.2)	6 847 (9.7)
	75–79	19 816 (14.0)	11 629 (13.8)	20 927 (14.0)	12 108 (13.9)	18 249 (15.3)	10 500 (14.9)
	80–84	31 517 (22.3)	18 010 (21.4)	33 370 (22.4)	18 698 (21.4)	27 515 (23.0)	15 447 (22.0)
	85–89	34 903 (24.7)	20 537 (24.4)	36 871 (24.7)	21 348 (24.4)	28 337 (23.7)	16 579 (23.6)
	90–94	23 524 (16.7)	14 001 (16.6)	24 856 (16.7)	14 490 (16.6)	17 339 (14.5)	10 225 (14.5)
	>94	7 460 (5.3)	4 931 (5.9)	7 859 (5.3)	5 092 (5.8)	4 688 (3.9)	3 225 (4.6)
Sex	Female	104 905 (74.3)	60 987 (72.5)	110 927 (74.3)	63 402 (72.5)	87 758 (73.5)	50 485 (71.8)
	Male	36 253 (25.7)	23 109 (27.5)	38 331 (25.7)	24 012 (27.5)	31 662 (26.5)	19 834 (28.2)
Pre-fracture	Outdoor mobility	86 719 (61.4)	63 293 (75.3)	92 021 (61.7)	65 861 (75.3)	83 780 (70.2)	57 616 (81.9)
Mobility	Indoor mobility	51 573 (36.5)	19 716 (23.4)	54 237 (36.3)	20 430 (23.4)	34 338 (28.8)	12 169 (17.3)
	No mobility	2 866 (2.0)	1 087 (1.3)	3 000 (2.0)	1 123 (1.3)	1 302 (1.1)	534 (0.8)
Pre-fracture	Home/sheltered housing	114 534 (81.1)	68 522 (81.5)	121 573 (81.5)	71 419 (81.7)	119 420 (100)	70 319 (100)
Residence	Nursing/residential care	26 624 (18.9)	15 574 (18.5)	27 685 (18.5)	15 995 (18.3)	0.0	0.0
Dementia	Yes	36 503 (25.9)	25 224 (30.0)	38 145 (25.6)	26 015 (29.8)	18 410 (15.4)	13 844 (19.7)
In-hospital death^a^		6 780 (4.8)	3 752 (4.5)	6 810 (4.6)	3 770 (4.3)	4 815 (4.0)	2 744 (3.9)
30-day death^b^		8 311 (5.9)	4 632 (5.5)	8 693 (5.8)	4 790 (5.5)	5 551 (4.6)	3 080 (4.4)
Change in residence^c^						4 461 (3.7)	2 712 (3.9)

^a^Among the development data set for model prediction of in-hospital death outcome, only 1 patient had missing 30 death outcomes and missing change in residence outcome. No missing data in the validation data set for this model.

^b^Among the development and validation data sets for the model prediction of 30-day death, 7 578 (5%) and 3 045 (4%) have missing in-hospital death outcome.

^c^Among the development and validation data sets for model prediction of change in residence outcome, 3 276 (3%) and 1 486 (2%) have missing in-hospital death outcome, respectively. No patient has missing 30 days death outcome.

^d^For the model prediction of change in residence outcome, study population includes only patients admitted from home/sheltered housing.

### Development and Internal Validation

#### In-hospital death

Among 141 158 patients, 6 780 (4.8%) died in-hospital, 72 401 (51.3%) were discharged, 48 798 (34.6%) were discharged to another unit, and 13 179 (9.3%) had stays longer than 30 days. Dementia and pre-fracture residence had nonconstant residuals across time indicating a potential violation to the proportional hazard assumption. However, further exploration of an interaction with time indicated no major violation for these predictors (Supplementary Figure 1).

The predicted risk of in-hospital death was calculated using the “*predict.crr*” function in the *cmprsk* package which uses the formula:


$$\begin{array}{l} 1-0.9909^{exp(LP_{1})}\;\;\; \end{array}$$

where 0.9909 is the baseline 30-day survival estimate and the linear predictor (LP_1_) is equal to


$$\begin{array}{l} \left(\beta_{1}\times age)+(\beta_{2}\times sex)+(\beta_{3}\times prefracture\;mobility\right)\\ +(\beta_{4}\times \;prefracture\;residence)+(\beta_{5}\times dementia)\;\;\; \end{array}$$

β _1_: 65–69 years: 0.3917426; 70–74 years: 0.6325601; 75–79 years: 0.8231233; 80–84 years: 1.2006534; 85–90 years: 1.5429587; 90–94 years: 1.8768660; 95 or more years: 2.2821073. β _2_: 0.6993337. β _3_: indoor: 0.6518865; no function: 0.7273951. β _4_: −0.1337233. β5: 0.1670263.

**Figure 2.**
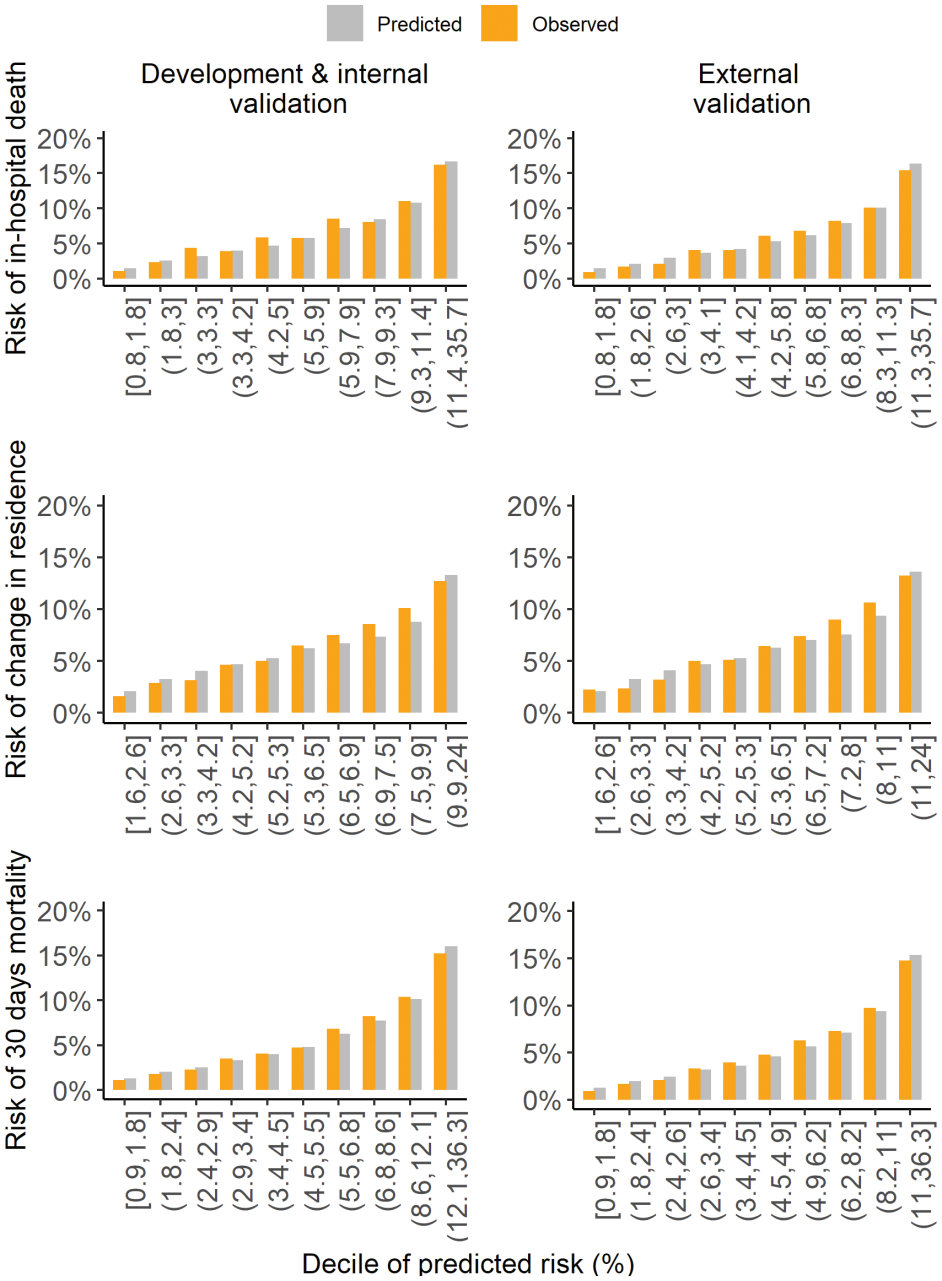
Calibration plots of predicted and observed risk of in-hospital death, change in residence, and 30-day death after hip fracture surgery for development and internal and external validation.

The model was well calibrated as evidenced by a calibration plot of predicted against observed risk across deciles of predicted risk (Figure 2), with an overall calibration measured by the O/E ratio of 0.90 (from 0.89 to 1.36 across risk deciles) with a weaker fit for those in the risk groups (3%–3.3%), (4.2%–5.2%), and (5.9%–7.9%). AUC and Brier scores were similar for the development and internal validation with optimism-adjusted AUC and Brier scores of 73.1% (95% CI, 72.6–73.7) and 5.66% (95% CI, 5.57–5.79), respectively (Table 2).

**Table 2.** Development, Internal Validation, and External (Temporal) Validation of Multivariable Prediction Models for In-hospital and 30-Day Death, and Change in Residence for Patients After Hip Fracture Surgery, Complete-Case Analysis

	Development, Internal Validation			External Validation		
	In-hospital Death	Change in Residence	30-Day Mortality	In-hospital Death	Change in Residence	30-Day Mortality
*n* events^a^(%)	6 780 (4.8)	4 461 (3.7)	8 693 (5.8)	3 752 (4.5)	2 712 (2.1)	4 790 (5.5)
30-day CIF % (95% CI)	7.1 (6.9–7.2)	6.2 (6.0–6.4)		5.9 (5.8–6.1)	6.4 (6.1–6.6)	
AUC^b^ % (95% CI)	72.9 (72.2–73.7)	71.4 (70.3–72.7)	71.1 (70.6–71.6)	73.1 (72.7–74.2)	71.7 (70.6–72.7)	71.2 (70.6–72.0)
Brier score^b^ % (95% CI)	5.7 (5.5–5.9)	5.6 (5.3–5.8)	5.3 (5.2–5.4)	5.3 (5.1–5.4)	5.7 (5.5–5.9)	5.0 (5.0–5.1)
Optimism-adjusted AUC % (95% CI)	73.1 (72.6–73.7)	71.5 (70.8–72.5)	71.1 (70.5–71.6)			
Optimism-adjusted Brier score % (95% CI)	5.7 (5.6–5.8)	5.6 (5.4–5.7)	5.3 (5.2–5.4)			

*Notes*: AUC = area under the curve; CI = confidence interval; CIF = cumulative incidence function.

^a^By 30 inpatient days for in-hospital death and change in residence.

^b^Apparent performance statistics.

The risk of in-hospital death was estimated at 32.0% for a man over the age of 94 years admitted from residential care with no pre-fracture mobility and a history of dementia. The risk of in-hospital death was estimated at 0.9% for a woman aged between 60 and 64 years admitted from home with outdoor mobility pre-fracture and no history of dementia.

#### Change in residence

Among 119 420 patients, 4 461 (3.7%) had a change in residence, 51 178 (42.9%) were discharged to their pre-fracture residence, 5 699 (4.8%) died in-hospital, 47 329 (39.6%) were discharged to another unit, and 10 753 (9.0%) had stays longer than 30 days. There was no evidence of violation to the proportional hazard assumption (Supplementary Figure 2).

The predicted risk of change in residence was calculated using the “*predict.crr”* function in the *cmprsk* package using the formula:


$$\begin{array}{l} 1-0.9800114{\;\;\;}^{exp(LP_{2})}\;\;\; \end{array}$$

where 0.9800114 is the baseline survival estimate for no change in residence and the linear predictor (LP_2_) is equal to


$$\begin{array}{ll} & (\beta_{6}*age)+(\beta_{7}*sex)+(\beta_{8}*prefracture\;mobility)+\\ & (\beta_{9}\;*dementia) \end{array}$$

β _6_: 65–69 years: 0.07259374; 70–74 years: 0.49701280; 75–79 years: 0.74251183; 80–84 years: 0.98564585; 85–90 years: 1.20026245; 90–94 years: 1.34492377; 95 or more years: 1.46828028. β _7_: −0.23046984. β _8_: Indoor:10857114; no function: 0.48041962. β _9_: 0.65983936.

The model displayed a tendency towards underfitting for those in the 7th to 9th risk deciles (6.5%–11.0%) as evidenced by a calibration plot of predicted against observed risk across deciles of predicted risk (Figure 2) with an overall calibration measured by the O/E ratio of 0.94 (range from 0.86 to 1.31 across risk deciles). AUC and Brier scores were similar for development and internal validation with optimism-adjusted AUC and Brier scores of 71.5% (95% CI, 70.8–72.5) and 5.5% (95% CI, 5.4–5.7) respectively (Table 2).

The risk of change in residence was estimated to be 24.0% for a woman over the age of 94 years with no pre-fracture mobility and a history of dementia. The risk of a change in residence was estimated to be 1.6% for a man aged between 60 and 64 years with outdoor mobility pre-fracture and no history of dementia.

#### 30-day death

Among 149 258 patients, 8 693 (5.8%) died by 30 days. VIFs indicated no collinearity (Supplementary Table 4). The standardized residual error presented in Supplementary Figure 3 revealed 16 data points (<0.5%) with absolute standardized residuals above 3 which were investigated as possible outliers.

The predicted risk of in-hospital death was calculated by the formula:


$$\begin{array}{l} \frac{1}{1+\;\;\;exp^{-LP_{3}}}\;\;\; \end{array}$$

where the linear predictor (LP_3_) is equal to


$$\begin{array}{ll} & -4.6851772\;+\;(\beta_{10}\times age)+(\beta_{11}\times sex)+\\ & (\beta_{12}\times prefracture\;mobility)+\\ & (\beta_{13}\times \;prefracture\;residence)+(\beta_{14}\times dementia)\; \end{array}$$

β _10_: 65–69 years: 0.3213738; 70–74 years: 0.4358315; 75–79 years: 0.6889942; 80–84 years: 0.9946787; 85–89 years: 1.3435684; 90–94 years: 1.6353948; 95 or more years: 2.0773348; β _11_: 0.7301712; β _12_: indoor: 0.6327345; no function: 0.7915023; β _13_: 0.1929848; β _14_: 0.3310316.

The model displayed no tendency to under- or overfitting as evidenced by a calibration plot of predicted against observed risk (Figure 2 and Supplementary Figure 4) with an overall calibration measured by the O/E ratio of 0.99 (ranged from 0.83 to 1.00 across risk deciles). AUC and Brier scores were similar for development and internal validation with optimism-adjusted AUC and Brier scores of 71.1% (95% CI, 70.6–71.6) and 5.30% (95% CI, 5.20–5.40), respectively (Table 2). An optimism of 1.00 (95% CI, 0.99–1.00) was identified for the calibration slope.

The risk of 30-day death was estimated to be 36.3% for a man over the age of 94 years admitted from residential care with no pre-fracture mobility and a history of dementia. The risk of in-hospital death was estimated to be 0.9% for a woman aged between 60 and 64 years admitted from home with outdoor mobility pre-fracture and no history of dementia.

#### Risk groups

Patients were clustered into low-, medium-, and high-predicted risk groups for each of the 3 outcomes using *K*-means clustering prior to assignment to mutually exclusive overall low (31% [*n* = 44 364]), medium (28% [*n* =39 542]), and high (41% [*n* =57 251]), risk across all 3 outcomes (Figure 1). Patients in the overall low-risk group were typically less than 80 years old with the majority female (66%), admitted from home (91%), with outdoor mobility pre-fracture (83%), and no dementia diagnosis (95%; Table 3). Compared to the overall low-risk group, a greater proportion of patients in the overall medium-risk group were older (94% aged 80 years or more), female (99%), and had a dementia diagnosis (14%; Table 3). Compared to the overall medium-risk group, patients in the high-risk group were of a similar age (94% aged 80 years or more); however, a greater proportion was male (37%), with indoor/no mobility (69%), admitted from nursing/residential care (40%) with a dementia diagnosis (50%; Table 3). The stratify-hip algorithm can be calculated online at: https://stratifyhip.co.uk. Nomograms for calculating the algorithm offline are available in Supplementary Figure 5.

**Table 3.** Characteristics of Patients Surgically Treated for Nonpathological First Hip Fracture According to the Overall Risk Group Based on Outcome-Driven Classification

		All	Low	Medium	High
		*N* = 141 157	*N* = 44 364	*N* = 39 542	*N* = 57 251
Age at admission (y)	60–64	4 618 (3.3)	4 590 (10.3)	9 (0.0)	19 (0.0)
	65–69	7 867 (5.6)	7 641 (17.2)	5 (0.0)	221 (0.4)
	70–74	11 453 (8.1)	10 447 (23.5)	576 (1.5)	430 (0.8)
	75–79	19 816 (14.0)	15 359 (34.6)	1 783 (4.5)	2 674 (4.7)
	80–84	31 517 (22.3)	5 933 (13.4)	17 937 (45.4)	7 647 (13.4)
	85–89	34 902 (24.7)	394 (0.9)	13 160 (33.3)	21 348 (37.3)
	90–94	23 524 (16.7)	0 (0.0)	6 072 (15.4)	17 452 (30.5)
	>94	7 460 (5.3)	0 (0.0)	0 (0.0)	7 460 (13.0)
Sex	Female	104 904 (74.3)	29 465 (66.4)	39 212 (99.2)	36 227 (63.3)
	Male	36 253 (25.7)	14 899 (33.6)	330 (0.8)	21 024 (36.7)
Pre-fracture	Outdoor mobility	86 719 (61.4)	36 895 (83.2)	34 390 (87.0)	15 434 (27.0)
Mobility	Indoor mobility	51 572 (36.5)	7 152 (16.1)	4 806 (12.2)	39 614 (69.2)
	No mobility	2 866 (2.0)	317 (0.7)	346 (0.9)	2 203 (3.8)
Pre-fracture	Home/sheltered housing	114 533 (81.1)	40 383 (91.0)	39 542 (100.0)	34 608 (60.4)
Residence	Nursing/residential care	26 624 (18.9)	3 981 (9.0)	0 (0.0)	22 643 (39.6)
Dementia	No	104 655 (74.1)	42 216 (95.2)	33 866 (85.6)	28 573 (49.9)
	Yes	36 502 (25.9)	2 148 (4.8)	5 676 (14.4)	28 678 (50.1)

### Sensitivity Analysis

For imputation results and where age was treated as a continuous predictor, summary performance statistics were comparable to performance estimates in the complete-case analysis. Full results of sensitivity analyses are available in Supplementary Tables 5 and 6.

### External (Temporal) Validation

Among the 90 102 patients in the validation data set, 84 096 (93%), 87 144 (97%), and 87 414 (97%) had complete data for predictors and in-hospital death, 30-day death, and change in residence, respectively. The majority were women, admitted from home, and were able to ambulate outdoors pre-fracture (Table 1). More than half were over 80 years of age and one-third had a diagnosis of dementia (Table 1).

#### In-hospital death

Among 84 096 patients, 3 752 (4.5%) died in-hospital, 44 348 (52.7%) were discharged, and 35 996 (42.8%) were censored by 30 days. Similar to the development data set, a weaker fit was observed for those in the risk groups (4.2%–6.8%; Figure 2). AUC and Brier scores were comparable to the development data set at 73.1% (95% CI, 72.7%–74.2%) and 5.26% (95% CI, 5.09%–5.43%), respectively.

#### Change in residence

Among 70 319 patients, 2 712 (2.1%) had a change in residence, 31 239 (24.6%) were discharged to their pre-fracture residence, 3 187 (2.5%) died in-hospital, and 33 181 (26.1%) were censored by 30 days. Similar to the development data set, the model displayed a tendency toward underfitting for those in the 7th–9th risk deciles (6.5%–11.0%; Figure 2). AUC and Brier scores were comparable to the development data set at 71.7% (95% CI, 70.6–72.7) and 5.7% (95% CI, 5.5–5.9), respectively.

#### 30-day death

Among 87 414 patients, 4 790 (5.5%) died by 30 days. Similar to the development data set, the model displayed no tendency to under- or overfitting as evidenced by a calibration plot of predicted against observed risk (Figure 2). AUC and Brier scores were comparable to the development data set at 71.2% (95% CI, 70.5%–0.71.9%) and 5.00% (95% CI, 4.95–5.13), respectively (Table 2).

#### Risk groups

The distribution of patients to overall low (33% [*n* = 27 566]), medium (31% [*n* = 25 669]), and high (36% [*n* = 30 861]) risk across outcomes and differences in the distribution of characteristics of patients across overall risk group were similar to the development data set (Supplementary Table 7).

## Discussion

### Main Findings

This study developed and validated multivariable prediction models for in-hospital death, 30-day death, and change in residence among 260 513 patients who underwent hip fracture surgery. The models enable the prediction of patients at low, medium, and high risk across the 3 outcomes. The models were well calibrated with acceptable discrimination between the groups consistently estimated during development and internal and external validation. The stratify-hip algorithm (comprised of 3 multivariable prediction models) and link to a freely available web-based app to facilitate risk prediction are provided.

### Comparison With Other Literature

Model performance measures for 30-day death were comparable between the current model and the Nottingham Hip Fracture Score (8). Similar to this score, older age, male sex, admission from a care home, and presence of dementia were predictive of 30-day death (8). The current model noted poorer pre-fracture mobility was also predictive of 30-day death. This predictor is not employed by the Nottingham Hip Fracture Score, which uses admission hemoglobin concentration, number of comorbidities, and malignancy as its final 3 predictors (8). A direct comparison of model performance was not possible due to the absence of admission hemoglobin from the current data set. For the current model, it was also noted pre-fracture mobility, together with age, sex, pre-fracture residence, and dementia, was predictive of time to in-hospital death and change in residence. Although the predictors selected for inclusion in the current models have previously been associated with poor outcomes (11–13), here an algorithm is provided for considering the prediction of these outcomes together at the point of admission. This outcome-driven stratify-hip algorithm generated distinct risk groups whereby a patient with a given set of predictors (collected at the point of admission) is allocated to 1 risk group.

### Interpretation

In the stratify-hip algorithm, the risk of short-term death was prioritized over changing residence. This “outcome-driven” approach was guided by an expectation the clinical needs of patients will vary according to which outcomes they are at risk of incurring. The approach mirrors the likely priority order of in-hospital care to reduce death risk and subsequently the risk of changing residence. This is potentially controversial for those admitted from home, given a study from 2000 indicated 80% of 194 women at risk of hip fracture indicated they would rather be dead than admitted to a care home (40). *K*-means clustering was considered for the overall group assignment. However, this approach does not generate “mutually exclusive” groups (a key requirement for employing the algorithm to inform a future-stratified approach) and the analysis would need to be on the same population for all 3 outcomes (but “changing residence” is only applicable to those admitted from home).

Given the weighting of the algorithm toward the high-risk group (Figure 1), it was surprising this group only constituted between 36% (external validation) and 41% (development) of the population. This is promising for the potential future clinical utility of the algorithm as it supports the hypothesis of distinct groups of patients within the population who may benefit from different care approaches. Indeed, the characteristics of patients exhibited an increased level of dependency from low- to high-risk groups. For example, those in the low-risk group were the youngest and had the lowest proportion with dementia whereas those in the high-risk group were the oldest and had the highest proportion with dementia. This was to be expected given those with greater dependency are more likely to be at risk of short-term death (and high-risk assignment).

### Future Research

Predictors were selected based on existing evidence and to optimize the simplicity of future implementation of the approach to risk stratification. Previously published models which incorporated more predictors yielded similar performance (7). The exception is the Orthopaedic Physiological and Operative Severity Score for the enUmeration of Mortality and Morbidity with a reported AUC of 83%; however, this score requires collection of physical (e.g., blood, electrocardiogram) and operative (blood loss) severity data limiting implementation. As the model performance was deemed acceptable with the five predictors identified, additional predictors were not explored here.

The type and intensity of clinical care may vary depending on the needs of an individual patient and organizational culture (3). The stratify-hip algorithm presented here is a first step in developing a stratified approach to care. The next step is to match risk groups to interventions tailored to their needs. Indeed, the risk groups likely benefit from differing in-hospital care (type, intensity, professional input) which may help to mitigate the demand–capacity mismatch for orthogeriatric and therapy input and reduce inconsistencies in prioritization (5,6). For example, patients in the high-risk group may require interventions that explicitly target risk factors for short-term death such as closer monitoring/managing perioperative medical complications (41), early mobilization (42), and/or consideration of end-of-life care (43). Conversely, those not at risk of short-term death but at risk of changing residence (medium-risk group) may be more appropriate for early-supported discharge (44). The goal of such an approach is to optimize outcomes across the entire population by ensuring equitable access to person-centered care, and not to ration care based on poorer prognosis. Any future-stratified approaches should be assessed for feasibility, acceptability, and effectiveness in randomized controlled trials (45).

Previous research highlighted challenges in communicating prognosis with patients, particularly in end-of-life care (46). These challenges were attributed to low confidence in prognostic estimates which was improved with the use of prediction models (46). The acceptability of the stratify-hip algorithm to patients, carers, and professional as a tool to support shared decision-making should be explored in future research. If deemed acceptable, the algorithm may be used by professionals to help set expectations for recovery in a timely manner with patients and their informal/formal carers.

The current study focused on short-term outcomes. It may be hypothesized short-term risk of death and/or changing residence may be related to the longer term risk of mobility loss, changing residence, and/or death. Future research may assess model performance in predicting longer-term outcomes, which in turn may inform community care.

### Limitations

Age was treated in 5-year increments and not as continuous with the intention of enabling paper-based implementation which may have led to a loss of power (47). However, similar model performance was noted when age was treated as continuously in a sensitivity analysis. Dementia was based on the absence or presence of a formal diagnosis code in hospital records which may be subject to underdiagnosis and subsequent misclassification in our models (48). However, the data source employed here recently had the highest reporting of dementia diagnoses across 3 U.K. data sources (48) and the ascertainment rate of 25%–30% is in keeping with the expected rate among older adults admitted to hospital with hip fracture (49). Date of death was not available limiting the ability to consider death at 30 days (inclusive of deaths after discharge) as a time-to-event outcome or to employ a multivariate analysis. There was a high proportion of right-censored observations for time-to-event outcomes due to discharges to other care settings. These observations are unlikely independent of the predictor–outcome association given patients admitted with no mobility may be less likely discharged to inpatient rehabilitation than those admitted with mobility pre-fracture. Groups were defined using *K*-means clustering algorithm which relies on researchers’ assumptions to a greater extent than other methods that rely on formal tests, for example, latent class analysis (50). However, *K*-means clustering, based on assumed 3 groups, led to the identification of 3 groups whose characteristics are likely amenable to different matched treatments which was the purpose of the algorithm. There is the potential for bias due to missing data. The sensitivity of the complete-case analysis to missingness was assessed by imputation which estimated similar performance. This was not surprising given imputed data sets were predominantly comprised of complete data (88%).

The stratify-hip algorithm may not be generalizable to settings where hospital stay varies from the average U.K. length of stay 15 days (5). Temporal external validation was completed and yielded a similar performance. Further external validation for more recent years (given implications of changes in social care funding for discharge destinations and in death rates over time), for different settings (given implications of health systems on outcomes), and using different definitions and/or measurements in similar patients is required to determine the international utility of the model.

## Conclusions

The current study details the new stratify-hip algorithm (comprised of 3 multivariable prediction models) enabling the identification of 3 distinct groups (low—31%, medium—28%, and high—41% risk) at differing risks of poor outcome after hip fracture surgery. The multivariable prediction models were well calibrated with acceptable discrimination during development and validation. Future research should seek to develop and test the feasibility and acceptability of the algorithm to group patients and match them to interventions. External validation beyond temporal validation is also recommended.

## Supplementary Material

glad053_suppl_Supplementary_files
