# Supplementary material for: Development and Validation of Multivariable Prediction Models for In-Hospital Death, 30-Day Death, and Change in Residence After Hip Fracture Surgery and the “Stratify-Hip” Algorithm
Source: J Gerontol A Biol Sci Med Sci. 2023 Feb 9;78(9):1659–68. doi: 10.1093/gerona/glad053 (PMC10460557; doi:10.1093/gerona/glad053)
Supplement: glad053_suppl_Supplementary_files [file glad053_suppl_supplementary_files.pdf]

SUPPLEMENTARY FILES

Table of contents

Figure S1: Schoenfeld residuals against days for each covariate in the model of in-hospital death outcome
Figure S2: Schoenfeld residuals against days for each covariate in the model of change in residence outcome
Figure S3: Plot of the standardized residuals for logistic regression model of 30 days death
Figure S4: Calibration plot for risk predictions from day 30 death model. Blue line: linear fit, red line: generalized additive model with integrated smoothness
Figure S5: Nomogram* visualizing regression models for (a) In-hospital death risk model[¥] (b) 30 days death risk model (c) change in residence risk model[¥] in patients with hip fracture
Table S1. Complete vs incomplete in the development dataset
Table S2. Complete vs incomplete in the external validation dataset
Table S3. Definition of change in residence
Table S4. Multicollinearity examination in the logistic model for 30 days death
Table S5: Imputation results
Table S6: Summary performance statistics for sensitivity analysis with age as a continuous predictor
Table S7. Characteristics of patients surgically treated for non-pathological first hip fracture according to the overall risk group based on outcome driven classification for the external validation dataset

Figure S1: Schoenfeld residuals against days for each covariate in the model of in-hospital death outcome

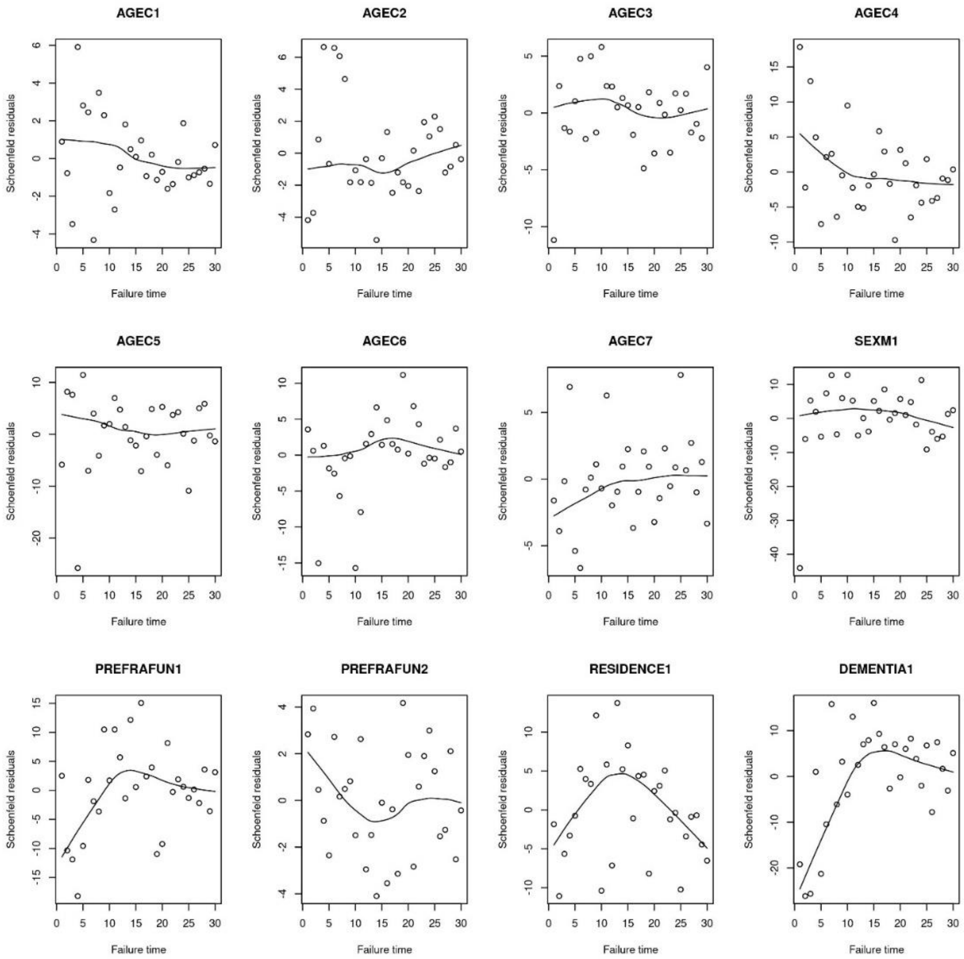

Figure S2: Schoenfeld residuals against days for each covariate in the model of change in residence outcome

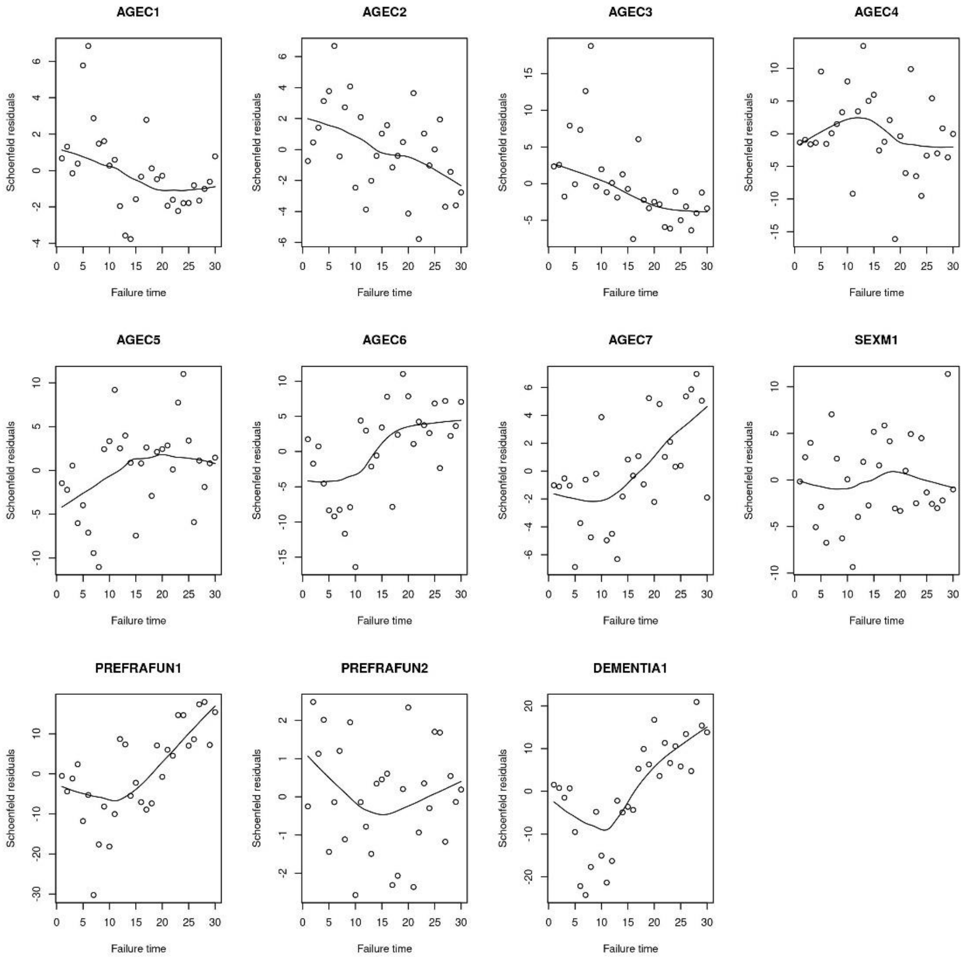

Figure S3: Plot of the standardized residuals for logistic regression model of 30 days death

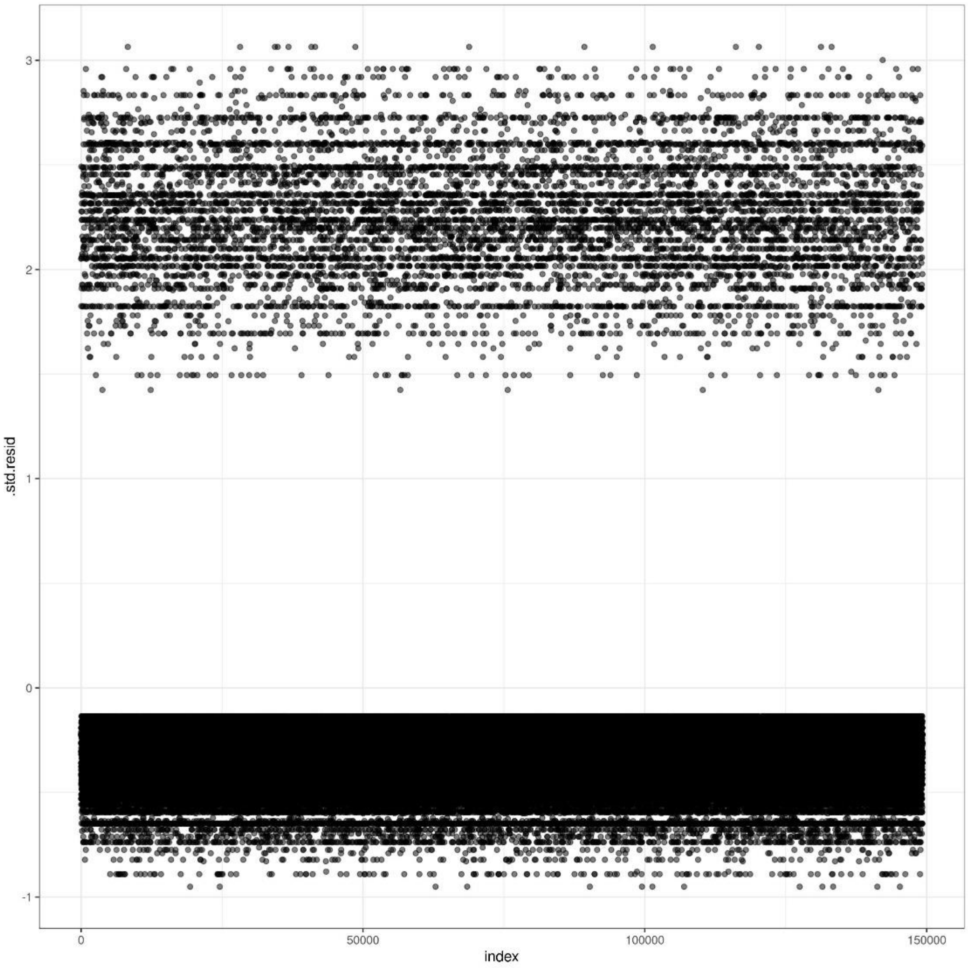

Figure S4: Calibration plot for risk predictions from day 30 death model. Blue line: linear fit, red line: generalized additive model with integrated smoothness

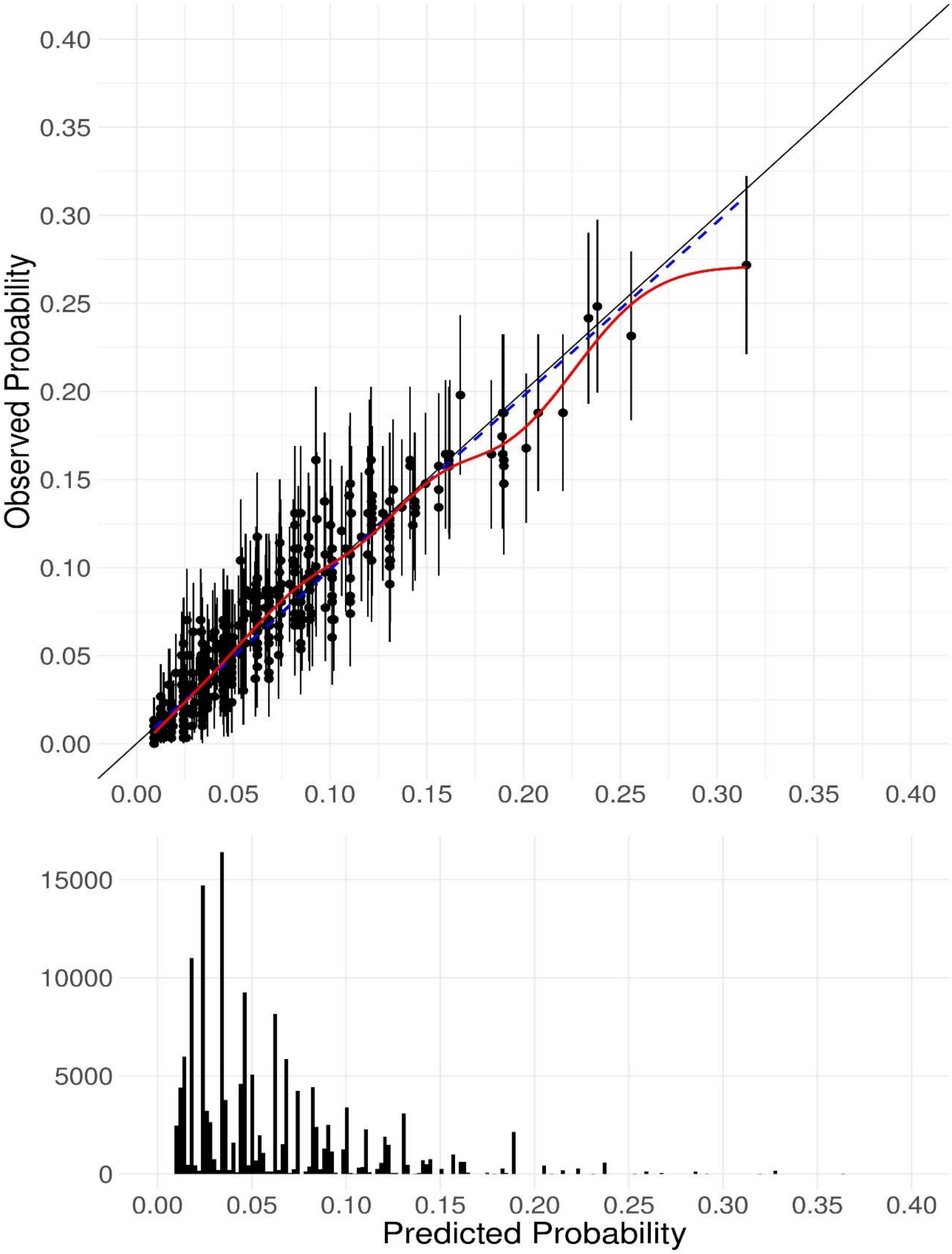

Figure S5: Nomogram* visualizing regression models for (a) In-hospital death risk model¥ (b) 30 days death risk model (c) change in residence risk model¥ in patients with hip fracture

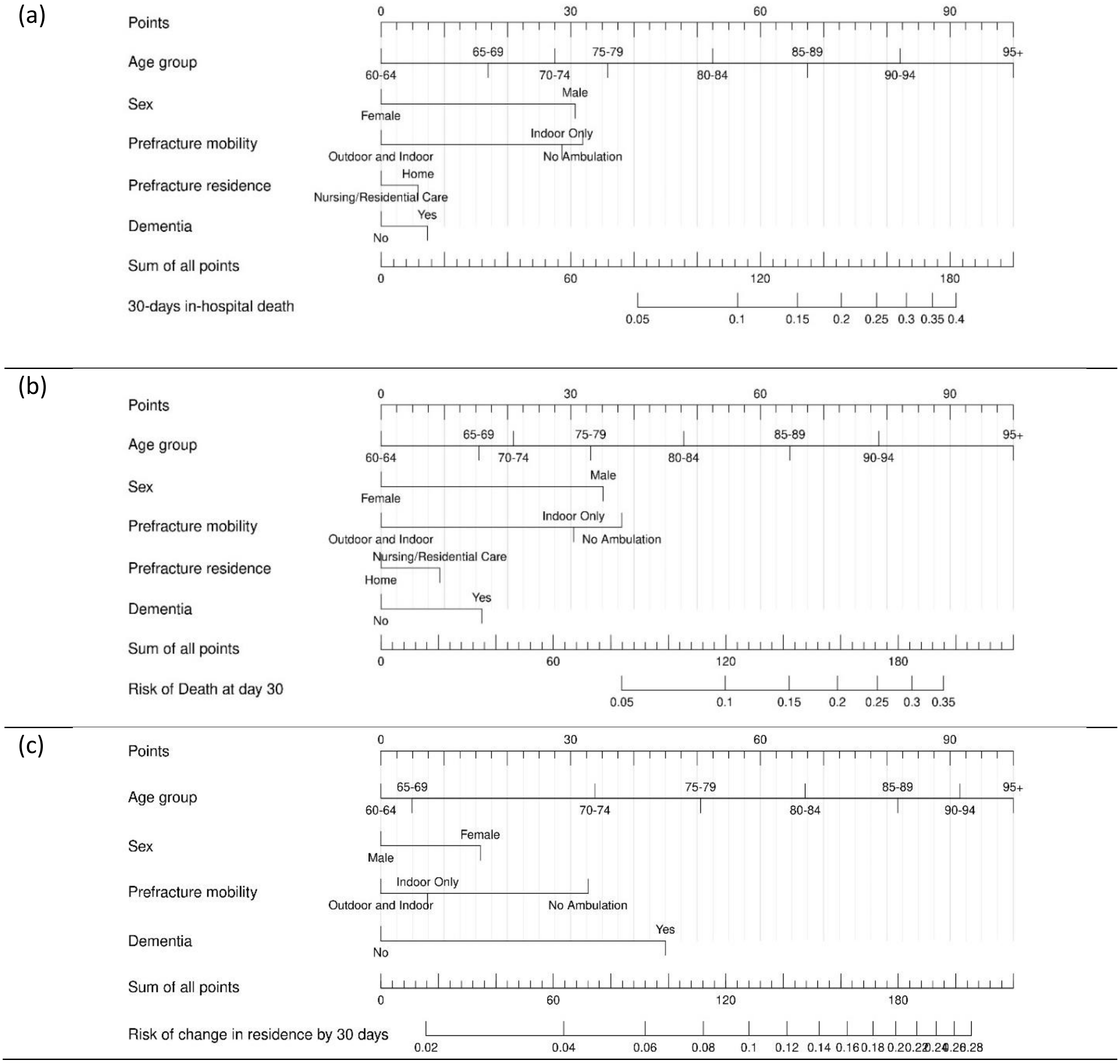

**To obtain nomogram predicted risk, locate patient values at each axis, Draw a vertical line to the "Point" axis to determine how many points are attributed for each variable value. Sum the points for all variables. Locate the sum on the "Total Points" line. Draw a vertical line towards the "risk of 30 days death", "risk of in-hospital by 30 days" and "" risk of change in residence by 30 days" axes to respectively determine the predicted risks for each of the three outcomes.*

¥In order to draw a nomogram in the presence of competing risks, a weighted data set was created for competing risks analyses [1].

1. Geskus RB. Cause-specific cumulative incidence estimation and the fine and gray model under both left truncation and right censoring. Biometrics 2011;67:39-49.

Table S1. Complete vs incomplete in the development dataset

		Complete Inhospital death outcome		Complete 30 days death outcome		Complete change in residence outcome	
		Incomplete	Complete	Incomplete	Complete	Incomplete	Complete
		N=19,239	N=141,158	N=21,152	N=149,258	N=7,581	N=119,420
Age	60-64	390 (2.0)	4618 (3.3)	467 (2.2)	4910 (3.3)	151 (2.0)	4616 (3.9)
	65-69	692 (3.6)	7867 (5.6)	783 (3.7)	8348 (5.6)	293 (3.9)	7711 (6.5)
	70-74	1146 (6.0)	11453 (8.1)	1320 (6.2)	12117 (8.1)	480 (6.3)	10965 (9.2)
	75-79	2337 (12.1)	19816 (14.0)	2644 (12.5)	20927 (14.0)	926 (12.2)	18249 (15.3)
	80-84	4229 (22.0)	31517 (22.3)	4679 (22.1)	33370 (22.4)	1649 (21.8)	27515 (23.0)
	85-89	5350 (27.8)	34903 (24.7)	5767 (27.3)	36871 (24.7)	2126 (28.0)	28337 (23.7)
	90-94	3714 (19.3)	23524 (16.7)	4015 (19.0)	24856 (16.7)	1395 (18.4)	17339 (14.5)
	>94	1311 (6.8)	7460 (5.3)	1398 (6.6)	7859 (5.3)	496 (6.5)	4688 (3.9)
	Missing	70 (0.4)	0 (0.0)	79 (0.4)	0 (0.0)	65 (0.9)	0 (0.0)
Sex	Female	13815 (71.8)	104905 (74.3)	15100 (71.4)	110927 (74.3)	5631 (74.3)	87758 (73.5)
	Male	5422 (28.2)	36253 (25.7)	6046 (28.6)	38331 (25.7)	1950 (25.7)	31662 (26.5)
	Missing	2 (0.0)	0 (0.0)	6 (0.0)	0 (0.0)	0 (0.0)	0 (0.0)
Prefracture mobility	Outdoor mobility	3197 (16.6)	86719 (61.4)	3687 (17.4)	92021 (61.7)	55 (0.7)	83780 (70.2)
	indoor mobility	3287 (17.1)	51573 (36.5)	3725 (17.6)	54237 (36.3)	5 (0.1)	34338 (28.8)
	No mobility	246 (1.3)	2866 (2.0)	284 (1.3)	3000 (2.0)	3 (0.0)	1302 (1.1)
	Missing	12509 (65.0)	0 (0.0)	13456 (63.6)	0 (0.0)	7518 (99.2)	0 (0.0)
Dementia	No	11334 (58.9)	104655 (74.1)	12523 (59.2)	111113 (74.4)	5604 (73.9)	101010 (84.6)
	Yes	7905 (41.1)	36503 (25.9)	8629 (40.8)	38145 (25.6)	1977 (26.1)	18410 (15.4)
Prefracture residence*	Own home/sheltered housing	7127 (37.0)	114534 (81.1)	7639 (36.1)	121573 (81.5)	7581 (100)	119,420 (100)
	Nursing/residential care	4108 (21.4)	26624 (18.9)	4337 (20.5)	27685 (18.5)		
	Missing	8004 (41.6)	0 (0.0)	9176 (43.4)	0 (0.0)		
Inhospital death				1675 (8.0)	6810 (4.6)	579 (7.6)	4815 (4.0)
	Missing			1729 (8.2)	7578 (5.1)	204 (2.7)	3276 (2.7)

30-day death		1808 (9.4)	8311 (5.9)			663 (8.7)	5551 (4.6)
	Missing	0	1 (< 1.0)			0	0
Change in residence		358 (1.9)	4461 (3.2)	359 (1.7)	4481 (3.0)		
	Missing	97 (1.0)	1 (< 1.0)	1196 (6.0)	4338 (3.0)		

*For the model prediction of change in residence outcome, study population includes only patients admitted from home/sheltered housing.

Table S2. Complete vs incomplete in the external validation dataset

		Complete Inhospital death outcome		Complete 30 days death outcome		Complete change in residence outcome	
		Incomplete	Complete	Incomplete	Complete	Incomplete	Complete
		N=2364	N=84096	N=2688	N=87414	N=682	70319
Age	60-64	46 (1.9)	2641 (3.1)	57 (2.1)	2772 (3.2)	10 (1.5)	2586 (3.7)
	65-69	109 (4.6)	5108 (6.1)	130 (4.8)	5346 (6.1)	32 (4.7)	4910 (7.0)
	70-74	168 (7.1)	7239 (8.6)	214 (8.0)	7560 (8.6)	52 (7.6)	6847 (9.7)
	75-79	289 (12.2)	11629 (13.8)	335 (12.5)	12108 (13.9)	73 (10.7)	10500 (14.9)
	80-84	495 (20.9)	18010 (21.4)	564 (21.0)	18698 (21.4)	135 (19.8)	15447 (22.0)
	85-89	617 (26.1)	20537 (24.4)	679 (25.3)	21348 (24.4)	136 (19.9)	16579 (23.6)
	90-94	393 (16.6)	14001 (16.6)	439 (16.3)	14490 (16.6)	99 (14.5)	10225 (14.5)
	>94	118 (5.0)	4931 (5.9)	130 (4.8)	5092 (5.8)	30 (4.4)	3225 (4.6)
	Missing	129 (5.5)	0 (0.0)	140 (5.2)	0 (0.0)	115 (16.9)	0 (0.0)
Sex	Female	1567 (66.3)	60987 (72.5)	1761 (65.5)	63402 (72.5)	481 (70.5)	50485 (71.8)
	Male	796 (33.7)	23109 (27.5)	926 (34.4)	24012 (27.5)	200 (29.3)	19834 (28.2)
	Missing	1 (0.0)	0 (0.0)	1 (0.0)	0 (0.0)	1 (0.1)	0 (0.0)
Prefracture mobility	Outdoor mobility	951 (40.2)	63293 (75.3)	1085 (40.4)	65861 (75.3)	98 (14.4)	57616 (81.9)
	indoor mobility	501 (21.2)	19716 (23.4)	564 (21.0)	20430 (23.4)	12 (1.8)	12169 (17.3)
	No mobility	41 (1.7)	1087 (1.3)	45 (1.7)	1123 (1.3)	4 (0.6)	534 (0.8)
	Missing	871 (36.8)	0 (0.0)	994 (37.0)	0 (0.0)	568 (83.3)	0 (0.0)
Dementia	No	1257 (53.2)	58872 (70.0)	1467 (54.6)	61399 (70.2)	468 (68.6)	56475 (80.3)
	Yes	1107 (46.8)	25224 (30.0)	1221 (45.4)	26015 (29.8)	214 (31.4)	13844 (19.7)
Prefracture residence*	Own home/sheltered housing	644 (27.2)	68522 (81.5)	715 (26.6)	71419 (81.7)	682 (100)	70319 (100)
	Nursing/residential care	296 (12.5)	15574 (18.5)	321 (11.9)	15995 (18.3)		
	Missing	1424 (60.2)	0 (0.0)	1652 (61.5)	0 (0.0)		
Inhospital death				193 (7.2)	3770 (4.3)	54 (7.9)	2744 (3.9)

	Missing			280 (10.4)	3045 (3.5)	26 (4.0)	1486 (2.1)
30-day death		220 (9.3)	4632 (5.5)			55 (8.1)	3080 (4.4)
	Missing	0	0			0	0
Change in residence		36 (1.5)	2712 (3.2)	37 (1.4)	2717 (3.1)		
	Missing	11 (< 1.0)	0	232 (8.6)	1610 (1.8)		

*For the model prediction of change in residence outcome, study population includes only patients admitted from home/sheltered housing.

Table S3. Definition of change in residence

status = 0 is censored (including if length of stay >30 days)
status = 1 Change in residence to higher level of care
status = 2 Discharge to prefracture residence
status = 3 Inhospital death
LOS = length of stay

Admit from	Discharge destination	Decision
Home	Home	if LOS <= 30 then status = 2, otherwise if LOS > 30 then status = 0, otherwise missing
Home	Nursing/residential care	if LOS <= 30 then status = 1, otherwise if LOS > 30 then status = 0, otherwise missing
Home	acute-hospital/rehab	if alive at 30 days, then right censor (status =0), if dead at 30 days, then status = 3, otherwise missing
Home	Missing	if alive at 30 days, then right censor (status =0), if dead at 30 days, then status = 3, otherwise missing
acute hospital/rehab	Home	if LOS <= 30 then status = 2, otherwise if LOS > 30 then status = 0, otherwise missing
Missing	Home	if LOS <= 30 then status = 2, otherwise if LOS >30 then status = 0 , otherwise missing
Home	Death	if LOS <= 30 then status = 3, otherwise if LOS > 30 then status = 0, otherwise missing

Table S4. Multicollinearity examination in the logistic model for 30 days death

	Df	VIF	VIF^(1/(2*Df))
Age	7	1.086132	1.005919
Sex	1	1.026793	1.013308
Prefracture mobility	2	1.217216	1.050369
Prefracture residence	1	1.430226	1.195921
DEMENTIA=Yes	1	1.357294	1.16503

Df: Degree of freedom. VIF: variance inflation factors. VIF^(1/(2*Df)) provides comparability among predictor sets having different dimension. Values more than 5 or 10 indicates a problematic amount of collinearity.

Table S5: Summary performance statistics following imputation of missing data

	Results from sensitivity analysis with imputed data		
	In-hospital death	Change in residence	30-day mortality
**Optimism adjusted AUC (95% CI)**	71.8 (71.3 – 72.3)	71.1 (70.3 – 71.8)	69.8 (69.3 – 70.3)
**Optimism adjusted Brier Score (95% CI)**	6.14 (6.02 – 6.26)	5.54 (5.39 – 5.69)	5.69 (5.60 – 5.79)

Table S6: Summary performance statistics for sensitivity analysis with age as a continuous predictor

	**Results from sensitivity analysis with age as continuous predictor**		
	In-hospital death	Change in residence	30-day mortality
**AUC (95% CI)**	73.2 (72.5 – 73.9)	71.9 (70.7 – 73.1)	71.2 (70.7 – 71.8)
**Brier Score (95% CI)**	5.7 (5.5 – 5.8)	5.6 (5.3 – 5.8)	5.3 (5.2 – 5.4)

Table S7. Characteristics of patients surgically treated for non-pathological first hip fracture according to the overall risk group based on outcome driven classification for the external validation dataset

		All	Low	Medium	High
		**N=84,096**	**N=27,566**	**N=25,669**	**N=30,861**
**Age at admission**	60-64	2641 (3.1)	2625 (9.5)	4 (0.0)	12 (0.0)
**(years)**	65-69	5108 (6.1)	4973 (18.0)	3 (0.0)	132 (0.4)
	70-74	7239 (8.6)	6600 (23.9)	415 (1.6)	224 (0.7)
	75-79	11629 (13.8)	8974 (32.6)	1482 (5.8)	1173 (3.8)
	80-84	18010 (21.4)	4017 (14.6)	10291 (40.1)	3702 (12.0)
	85-89	20537 (24.4)	377 (1.4)	9358 (36.5)	10802 (35.0)
	90-94	14001 (16.7)	0 (0.0)	4116 (16.0)	9885 (32.0)
	>94	4931 (5.9)	0 (0.0)	0 (0.0)	4931 (16.0)
**Sex**	Female	60987 (72.5)	17671 (64.1)	25264 (98.4)	18052 (58.5)
	Male	23109 (27.5)	9895 (35.9)	405 (1.6)	12809 (41.5)
**Prefracture mobility**	Outdoor mobility	63293 (75.3)	25036 (90.8)	23986 (93.4)	14271 (46.2)
	indoor mobility	19716 (23.4)	2413 (8.8)	1546 (6.0)	15757 (51.1)
	No mobility	1087 (1.3)	117 (0.4)	137 (0.5)	833 (2.7)
**Prefracture**	Home/sheltered housing	68522 (81.5)	24560 (89.1)	25669 (100.0)	18293 (59.3)
**residence**	Nursing/residential care	15574 (18.5)	3006 (10.9)	0 (0.0)	12568 (40.7)
**Dementia**	No	58872 (70.0)	25441 (92.3)	20358 (79.3)	13073 (42.4)
	Yes	25224 (30.0)	2125 (7.7)	5311 (20.7)	17788 (57.6)
